# Cloning and functional analysis of the juvenile hormone receptor gene *CsMet* in *Coccinella septempunctata*

**DOI:** 10.1093/jisesa/ieae065

**Published:** 2024-07-03

**Authors:** Ying Cheng, Yuhang Zhou, Cao Li, Jianxue Jin

**Affiliations:** Insect Research Group, Institute of Plant Protection, Guizhou Provincial Academy of Agricultural Sciences, Guiyang, China; Insect Research Group, Institute of Plant Protection, Guizhou Provincial Academy of Agricultural Sciences, Guiyang, China; Insect Research Group, Guizhou Provincial Pollution-free Engineering Center of Plant Protection, Guiyang, China; Insect Research Group, Institute of Plant Protection, Guizhou Provincial Academy of Agricultural Sciences, Guiyang, China

**Keywords:** ladybug, methoprene-tolerant, clone, expression, reproductive regulation

## Abstract

The potential role of the juvenile hormone receptor gene (*methoprene-tolerant*, *Met*) in reproduction of *Coccinella septempunctata* L. (Coleoptera: Coccinellidae)*(Coleoptera: Coccinellidae),* was investigated by cloning, analyzing expression profiles by quantitative real-time PCR, and via RNA interference (RNAi). *CsMet* encoded a 1518-bp open reading frames with a predicted protein product of 505 amino acids; the latter contained 2 Per-Arnt-Sim repeat profile at amino acid residues 30–83 and 102–175. *CsMet* was expressed in different *C. septempunctata* larvae developmental stages and was most highly expressed in third instar. *CsMet* expression in female adults gradually increased from 20 to 30 d, and expression levels at 25 and 30 d were significantly higher than levels at 1–15 d. *CsMet* expression in 20-d-old male adults was significantly higher than in males aged 1–15 d. *CsMet* expression levels in fat body tissues of male and female adults were significantly higher than expression in the head, thorax, and reproductive system. At 5 and 10 d after *CsMet*-dsRNA injection, *CsMet* expression was significantly lower than the controls by 75.05% and 58.38%, respectively. Ovary development and vitellogenesis in *C. septempunctata* injected with *CsMet*-dsRNA were significantly delayed and fewer mature eggs were produced. This study provides valuable information for the large-scale rearing of *C. septempunctata*.

## Introduction

Juvenile hormone (JH) is one of the most important hormones in regulating insect development, metamorphosis, and reproduction (Shelby et al. 2007, Riddiford et al. 2010, Hiruma and Kaneko 2013). In female adults, JH can promote fat body synthesis, vitellogenin (Vg) secretion, and ovarian absorption of Vg (Liu et al. 2019a, Han et al. 2022). In male adults, JH promotes the growth, development, and maturation of reproductive glands, and the production of glandular secretions (Adnan et al. 2020, Zhang 2021). With respect to JH-regulated insect reproduction, the methoprene-tolerant (*Met*) signaling pathway plays an important role in modulating ovary development, egg formation, embryonic development, and mating behavior (Roy et al. 2018, Peng et al. 2019, Wu et al. 2021, Han et al. 2022). RNAi-mediated silencing of *Met* in other insects, namely, *Chilo suppressalis*, *Sogatella furcifera*, and *Schistocerca gregaria* resulted in reduced transcription of *Vg* and the gene encoding vitellogenin receptor (*VgR*); furthermore, ovary development was delayed and egg production was significantly reduced (Gijbels et al. 2019, Hu et al. 2019, Miao et al. 2020). In male fruit flies lacking a functional *Met*, the physiological effects of JH were weakened, and protein accumulation in male accessory glands was reduced (Wilson et al. 2003). Furthermore, the injection of JH into newly emerged male adults of *Agrotis ipsilon* induced *Met* transcription, which increased the length and protein content of male accessory glands; in contrast, RNAi-mediated silencing of *Met* reduced the length and protein content of male accessory glands (Gassias et al. 2021).

The ladybug, *Coccinella septempunctata* L. (Coleoptera: Coccinellidae), is an important natural enemy of aphids, whiteflies, and jassids. After consuming an artificial diet, egg production and hatching rates decrease in *C. septempunctata*, which restricts large-scale production of lady beetle. Using transcriptome sequencing, our research team demonstrated that the expression of the JH receptor *Met* was downregulated in *C. septempunctata* fed on an artificial diet (Cheng et al. 2020a). We speculated that the decline in egg production and hatching rates of *C. septempunctata* reared on artificial diets may be caused by downregulation of *Met* expression. When JH is added to artificial diet, it can significantly improve both egg production and hatching rates (Cheng et al. 2023); however, the spatio-temporal expression of *Met* and regulation of reproduction by *Met* in *C. septempunctata* are unclear. In the present study, we utilized the transcriptome database of *C. septempunctata* to clone the full-length cDNA sequence of *Met*. The expression profile of *Met* was analyzed in different developmental stages and tissues, and RNAi was utilized to evaluate the role of *Met* in *C. septempunctata* reproduction. The results provide new insights into the role of the JH signaling pathway in regulating ladybug reproduction and are useful in the context of large-scale rearing of this important natural enemy.

## Materials and Methods

### Insects

*C. Septempunctata* were collected in wheat fields. Ladybugs were reared indoors on *Aphis craccivora* Koch (Hemiptera: Aphididae) for over 20 generations. Experiments were performed in artificial climate chambers at 25 ± 1 °C, 70 ± 5% RH with a 16:8 h light: dark photoperiod.

### Reagents

The reagents and kits used in this study include the following: the Revert Aid First Strand cDNA Synthesis Kit (Fermentas Co.); Eastep Super Total RNA Isolation Kit and pGEM-T Easy Vector (Promega Co.); 3ʹRACE SMARTer RACE cDNA Amplification Kit (Clontech Co.); 5ʹRACE System for Rapid Amplification of cDNA Ends (Invitrogen Co.); IScript cDNA Synthesis Kit and iTaq Universal SYBR Green Supermix Kit (Bio-Rad Co.); Phanta Max Super-Fidelity DNA Polymerase and Fast Pure Gel DNA Extraction Mini Kit (Vazyme Co.); and Transcript Aid T7 High Yield Transcription Kit (Thermo Co.).

### Instruments

The equipment used in this study included the following: DYY-6C power supply and WD-9403F gel imaging system (Beijing Liuyi Instrument Factory); BIOMATE3S nucleic acid protein analyzer (Thermo Fisher Co.); and T-100 PCR gradient thermal cycler and CFX96 real-time PCR system (Bio-Rad Co.); microinjector Femtojet 4i (Eppendorf).

### RNA Extraction and cDNA Synthesis

Ten-day-old female adults were grinded to a powder in liquid nitrogen and then transferred to 1.5 ml RNase-free microcentrifuge tubes. Trizol (1 ml) was added, and the mixture was incubated for 5 min at room temperature; trichloromethane (200 μl) was then added, gently mixed, incubated at room temperature for 3 min, and then centrifuged at 12,000× *g* for 15 min at 4 °C. A 600 μl volume of the supernatant was transferred into a new microcentrifuge tube, 500 μl of 100% isopropanol was added, and the mixture was incubated at room temperature for 10 min. The suspension was then centrifuged at 12,000× *g* at 4 °C for 10 min; the supernatant was then removed and 75% ethanol was added, gently inverted eight times, and centrifuged at 7,500× *g* for 5 min at 4 °C. Ethanol was then removed and RNA pellets were allowed to air dry for 5–10 min. RNA (1 μg) was used as a template, and the first strand of cDNA was synthesized using the Revert Aid First Strand cDNA Synthesis Kit and stored at −80 °C for future use.

### Cloning and Sequencing of *Met*

#### Primer design

The *Met* sequence was identified in the transcriptome database of *C. septempunctata* constructed in our laboratory. Primers were designed using ClustalX and Primer Premier 5.0 software, and primer synthesis was conducted by Sangon Biotech Co. (Shanghai) (Table 1).

**Table 1.** Primers used in this study (5ʹ–3ʹ)

Primer name	Primer sequence	Usage
Met-MF Met-MR	AAATGTSAAYTCCTTGTCGT CCHCKRTCRTACATYTGTCT	Intermediate fragments
Met-3ʹ1 Met-3ʹ2 Met-5ʹ1 Met-5ʹ2	TGTGGGTAACGACATTTGGCTGCT GTGGACGGTCGTATTGTCTGTTGC CCATCTTTGCGAGTTCTC ACGTCTCAGCTTCTCCGC	RACE
Met-QF Met-QR	GGGTGAGAGTGATGAGCGTT GCAGCCAAATGTCGTTACCC	Real-time PCR
Met-dsDNA-F	taatacgactcactatagggGATGAATCGACCGGAAAAGA	dsRNA synthesis
Met-dsDNA-R	taatacgactcactatagggAGCAAGGAGACGACGGTAGA	
GFP-dsDNA-F	taatacgactcactatagggGCCAACACTTGTCACTACTT	
GFP-dsDNA-R	taatacgactcactatagggGGAGTATTTTGTTGATAATGGTCTG	
Actin-F Actin-R	GATTCGCCATCCAGGACATCTC TCCTTGCTCAGCTTGTTGTAGTC	

#### Amplification of intermediate fragments

Using female cDNA as a template, PCR amplification of intermediate sequences was performed using Met-MF and Met-MR primers (Table 1). The reaction was conducted in a 50 μl volume and contained the following components: PCR-grade H_2_O (15 μl); 2X Ex Taq buffer (25.0 μl, Takara); 10mMdNTP mix (1.0 μl); Ex Taq (1.0 μl, Takara); cDNA, 5.0 μl; and Met-MF/MR primers, 1.5 μl. The reaction protocol included a 2-min denaturation at 94 °C; 94 °C for 30 s, 55 °C for 30 s, 72 °C for 1 min, 35 cycles; and 72 °C for 10 min. PCR products were analyzed on 1.0% agarose gels. The OMEGA kit was used to recover target fragments as recommended by the manufacturer.

#### 3ʹ and 5ʹ RACE PCR

Using the 3ʹ and 5ʹ first strand cDNA as templates, full-length amplification of *Met* was performed using the SMARTer RACE cDNA Amplification Kit and the 5ʹ RACE System for Rapid Amplification of cDNA Ends. PCR products were purified and recovered by 1% agarose gel electrophoresis, and then cloned to pmd18-T carrier (pmd18-T Vector Cloning Kit, TaKaRa 6011), transformed DH5a competent cells, and sequenced by Sangon Biotech Co. The open reading frames (ORF) Finder program (https://www.ncbi.nlm.nih.gov/orffinder/) was used to identify the coding region for *Met*, and the ExPASy program (https://web.expasy.org/computepi/) was used to identify the isoelectric point and molecular weight of the *Met*-encoded protein. SMART 8 software (https://prosite.expasy.org/) was used to identify the structural domain of the encoded protein.

#### *Alignment and phylogenetic analyses of* Met

The NCBI BLAST program (https://blast.ncbi.nlm.nih.gov/) was used to find related sequences between *C. septempunctata Met* (*CsMet*) and orthologs in other insect species. To further investigate evolutionary relationships, the amino acids of closed related insect *Met* sequences were downloaded from NCBI, and a phylogenetic tree was constructed using the neighbor-joining method by MEGA 6 software.

#### *Spatio-temporal expression of* CsMet

Samples of different developmental stages (e.g., 2-d-old eggs, first- to fourth-instar larvae, pupae, and 1-, 5-, 10-, 15-, 20-, 25-, and 30-d-old female and male adults) were collected along with heads, thorax, fat body, and reproductive systems of 10-d-old female and male adults. Each sample was biological replicated 3 times in per developmental stage. A single replicate consisted of the following: 60 eggs; 30, first-instar larvae; 15, second-instar larvae; 10, third-instar larvae; 4, fourth-instar larvae; 4 pupae; and 4 adults. The head, thorax, fat body, and reproductive system were dissected from six 10-d-old female and male adults, respectively, and considered as one biological replicate. Collected samples were immediately frozen in liquid nitrogen and stored at −80 °C for future use.

Total RNA was extracted from each sample according to the instructions included with the Eastep Super Total RNA Isolation Kit. The iScript cDNA Synthesis Kit was used to reverse transcribe and synthesize cDNA, and samples were stored at −20 °C for future use. The Met-specific primers, Met-QF/Met-QR, and Actin-F/Actin-R (internal standard, Liu et al. 2019b) were used to measure expression in different developmental stages and tissues (Table 1). qPCR was conducted in a 20 μl volume containing the following: cDNA template, 2 μl; forward and reverse primers, 2 μl each; Sso Advanced Universal SYBR Green Supermix, 10 μl; and ddH_2_O, 4 μl. The qPCR reaction conditions included pre-denaturation at 95 °C for 2 min; 95 °C denaturation for 5 s; and 60 °C annealing and extension for 30 s for a total of 39 cycles. The relative expression level of *Met* was calculated using the 2^−∆∆ Ct^ method (Pfaffl 2001).

### RNAi of *CsMet*

#### dsRNA synthesis

Full-length *CsMet* were used to design *CsMet*-dsRNA primers, and T7 promoters were incorporated into primer ends (Table 1). The gene encoding green fluorescent protein (GFP) was used as a control, and GFP-specific primers are shown in Table 1. The Phanta Max Super-Fidelity DNA Polymerase Kit was used to amplify *CsMet* and *GFP*. PCR products were separated by electrophoresis, isolated with the FastPure Gel DNA Extraction Mini Kit, and then in vitro transcription with the Transcript Aid T7 High Yield Transcription Kit to synthesize *CsMet*-dsRNA and GFP-dsRNA. The reaction for in vitro transcription contained 6 μl DEPC-treatedwater, 8 μl 5 × TranscriptAid reaction buffer, 16 μl dNTPs, 6 μl template DNA (620 ng/μl), and 4 μl of Transcript Aid enzymemix. The reaction was conducted at 37 °C for 4 h, this was followed by treatment with DNase I (2 μl) with incubation at 37 °C for 15 min, and a final treatment with 0.5M EDTA (2 μl, pH 8.0) and incubation at 65 °C for 10 min. Reactions were stored at −80 °C until needed.

#### dsRNA injection

Selected 1-d-old female adults were microinjected with 1 μl *Met*-dsRNA (4,500 ng/μl). Female adults were placed in a 25 ml jar and exposed to CO_2_ gas; anaesthetized females were then injected with dsRNA at the internodes separating the third and fourth abdominal segments. Microinjection needles were inserted for 5 s and then removed. Controls consisted of the *GFP*-dsRNA-injected group and another group that was not injected. Each treatment contained 50 females, and all experiments were repeated 3 times.

#### Reproductive function analysis of dsRNA-treated females

RNA extraction, cDNA synthesis, and RT-qPCR were performed on female adults on the fifth and tenth days after dsRNA injection using methods outlined in Sections 2.4 and 2.7. Each treatment was 3 samples, and each sample contained 4 females. Ovaries were dissected on the fifth and tenth days after injection and viewed with a stereomicroscope equipped with Image View software; the latter was used to measure the length and width of ovaries. Each treatment contained 30 dissected females. After injection, 10 females were paired with males emerging on the same day, and each treatment was replicated 3 times for a total of 30 pairs. Egg numbers were recorded daily for 30 d. The control group consisted of noninjected females.

### Statistical Analysis

One-way ANOVA was performed on the experimental data, and the multiple comparison LSD method was used to determine significance with DPS 19.05 software (Tang and Zhang 2013).

## Results

### Cloning and Sequence Analysis of *C. septempunctata Met*

**Fig. 1.**
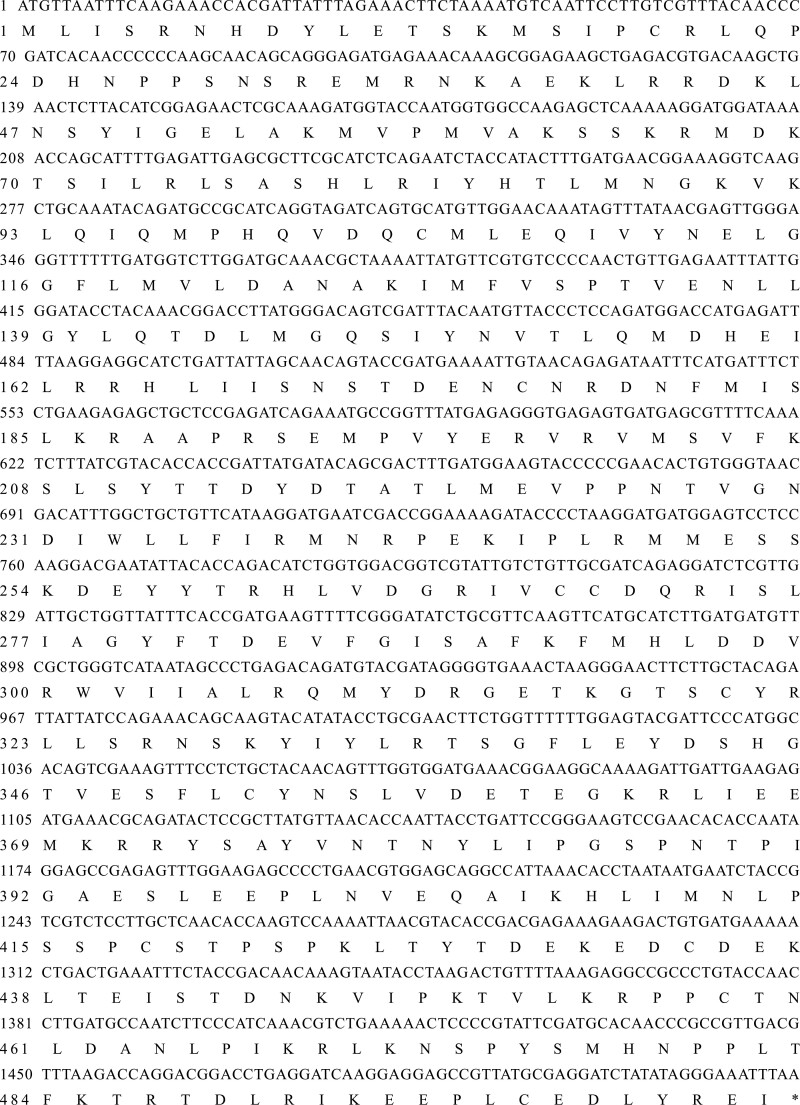
Nucelotide and deduced amino acid sequence of *CsMet.*

Based on our transcriptome data of *C. septempunctata*, cDNA from female adults was used as a template to clone *CsMet* (GenBank accession no. OR135688). Sequence analysis showed that *CsMet* cDNA was 1984 bp and encoded a 1518 bp ORF consisting of 505 amino acids and 5ʹ and 3ʹ noncoding regions of 340 and 126 bp, respectively (Fig. 1). The molecular weight of the CsMet protein was 58.48 kD, and its isoelectric point was 8.15. There were 3 conserved regions, namely, a basic helix-loop-helix (bHLH) region and a Per-Arnt-Sim (PAS) repeat, at amino acid residues 30–83 and 102–175, respectively.

### Alignment and Phylogenetic Analysis of CsMet

**Fig. 2.**
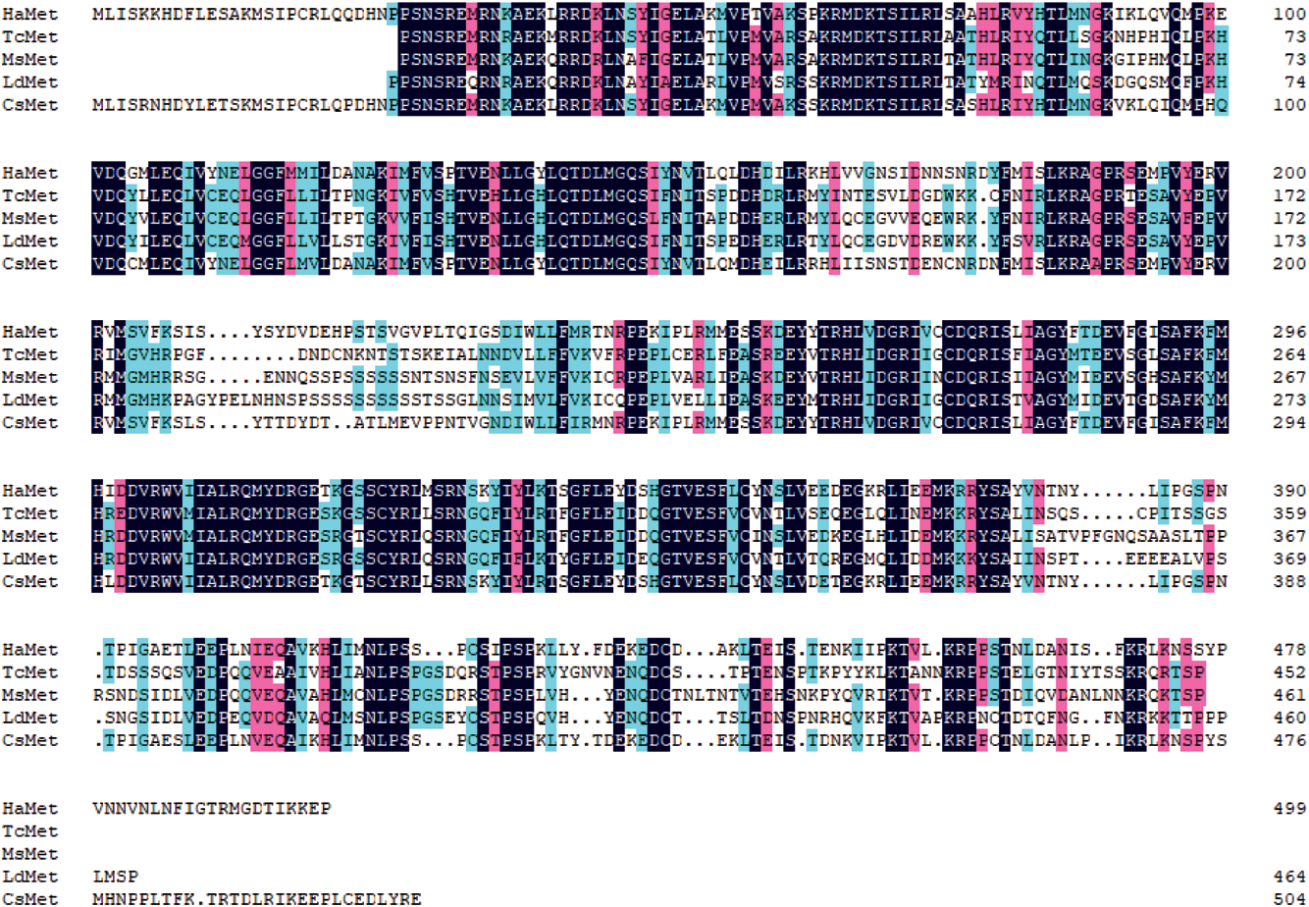
Multiple alignment of CsMetin *Coccinella septempunctata* and orthologs from other insect species. Abbreviations: HaMet, *Harmonia axyridis*; TcMet: *Tribolium castaneum*; MsMet, *Monochamus saltuarius*; LdMet, *Leptinotarsa decemlineata*; and CsMet, *Coccinlla septempunctata*.

**Fig. 3.**
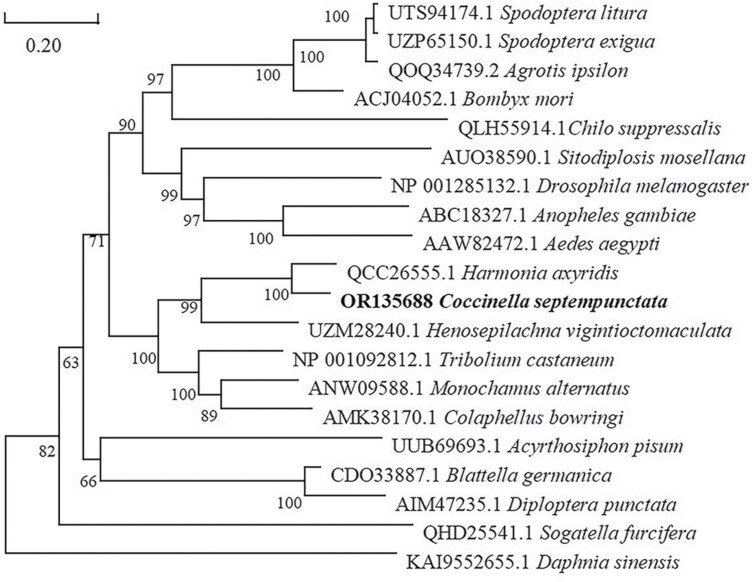
Phylogenetic tree of CsMet in *Coccinlla septempunctata* and orthologs from other insects.

Multiple alignment (DNAMAN 6.0.3) analysis of CsMet revealed 84.17% similarity with HaMet in *Harmonia axyridis* and 56.36%, 54.39%, and 51.70% identity with TcMet, MsMet, and LdMet from *Tribolium castaneum*, *Monochamus saltuarius*, and *Leptinotarsa deceminineata*, respectively (Fig. 2). Phylogenetic analysis indicated that CsMet and HaMet clustered together in one branch (Fig. 3), which was consistent with identification based on entomology.

### Spatiotemporal Expression Analysis of *CsMet*

**Fig. 4.**
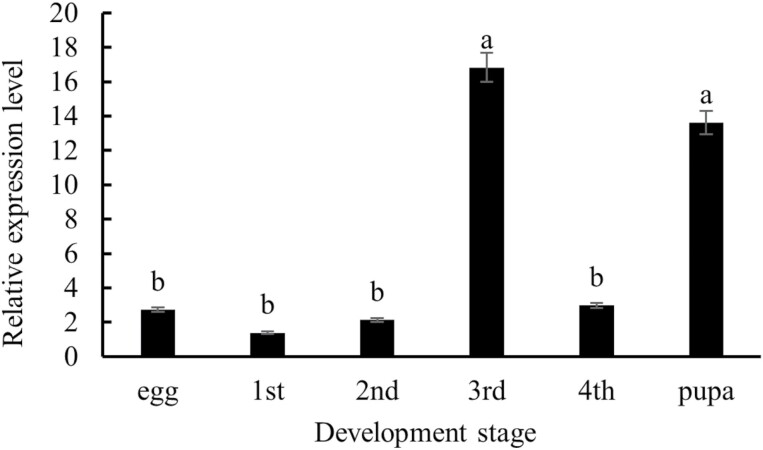
Relative expression levels of *CsMet* in different developmental stages of *Coccinella septempunctata.* Data are means ± SD. Columns labeled with different letters indicate significance at *P* < 0.05 using the LSD test.

There were significant differences in expression levels of *CsMet* among developmental stages (*F* = 13.21, *df* = 5,10, *P* = 0.0004) (Fig. 4). *CsMet* expression level was low in eggs and first-, second-, and fourth-instar larvae; however, expression in third-instar larvae was 12.10-fold higher than first-instar larvae (Fig. 4). Expression in the pupal stage (2-d-old) was upregulated at levels 9.80-fold higher than first-instar larvae.

**Fig. 5.**
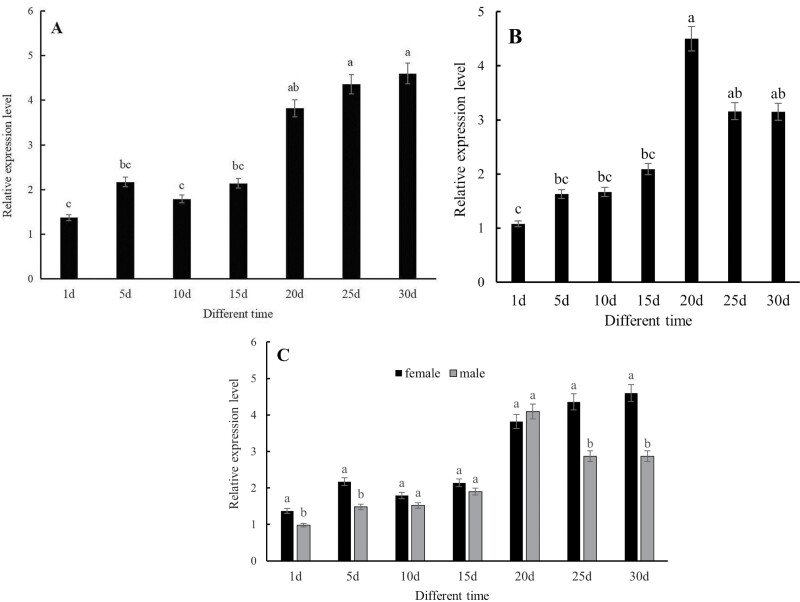
Relative expression levels of *CsMet* in different stages of *Coccinella septempunctata* adults. Panels A) female adults; B) male adults; and C) female and male adults. Data are means ± SD. Columns labeled with different letters indicate significance at *P* < 0.05 using the LSD test.

There was a significant difference in *CsMet* expression among various ages of *C. septempunctata* female adults (*F* = 4.10, *df* = 6,12, *P* = 0.018) (Fig. 5A). Expression was relatively low in females that were 1-, 5-, 10-, and 15-d-old; however, expression began to increase at 20 d and was highest in 30-d-old females where levels were 3.36-fold higher than that in 1-d-old females. *CsMet* was also differentially expressed in the various age male adults (*F* = 3.51, *df* = 6,12, *P* = 0.031) (Fig. 5B). Expression levels in male adults that were 1-, 5-, 10-, and 15-d-old were lower than levels at 20–30 d and were highest in 20-d-old male adults, where levels were 4.12-fold higher than in 1-d-old males. With the exception of levels in 20-d-old adults, *CsMet* expression in female adults was higher than male adults (Fig. 5C).

**Fig. 6.**
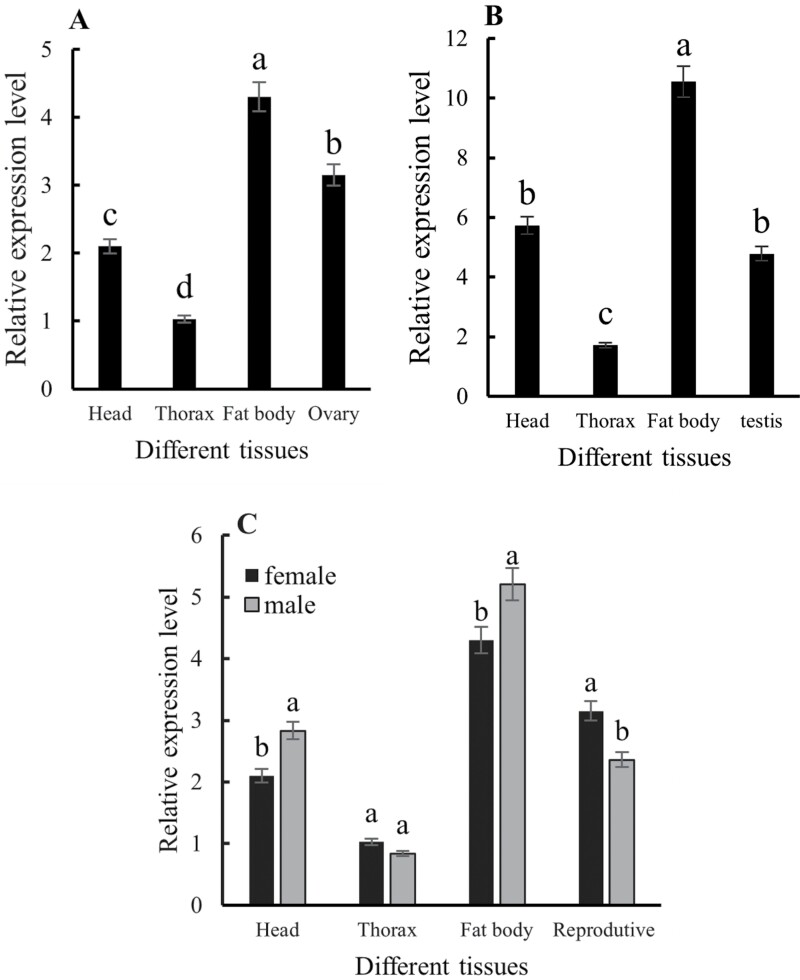
Relative expression levels of *CsMet* in different tissues of *Coccinella septempunctata* adults. Panels A) female adults; B) male adults; and C) female and male adults. Data are mean ± SD. Columns labeled with different letters indicate significance at *P *< 0.05 using the LSD test.

There were significant differences in *CsMet* expression among different tissues of female adults (*F* = 22.61, *df* = 3,6, *P* = 0.001) (Fig. 6A). *CsMet* was expressed in all tissues of female adults with the greatest levels in fat body, which were significantly higher than in the head, thorax, and ovaries. Similarly, *CsMet* expression also varied in different tissues of male adults (*F* = 24.68, *df* = 3,6, *P* = 0.001), and the highest level was observed in fat body tissue, which was 6.17-fold higher than in the thorax (Fig. 6B). Expression levels in the head and testes were not significantly different from each other, but were significantly higher than thorax. *CsMet* expression in the head and fat body of males was significantly higher than levels in females, whereas *CsMet* expression levels in the thorax and reproductive system were lower in males as compared to females (Fig. 6C).

### Silencing of *CsMet*

#### *Effect of dsRNA injection on* CsMet *expression*

**Fig. 7.**
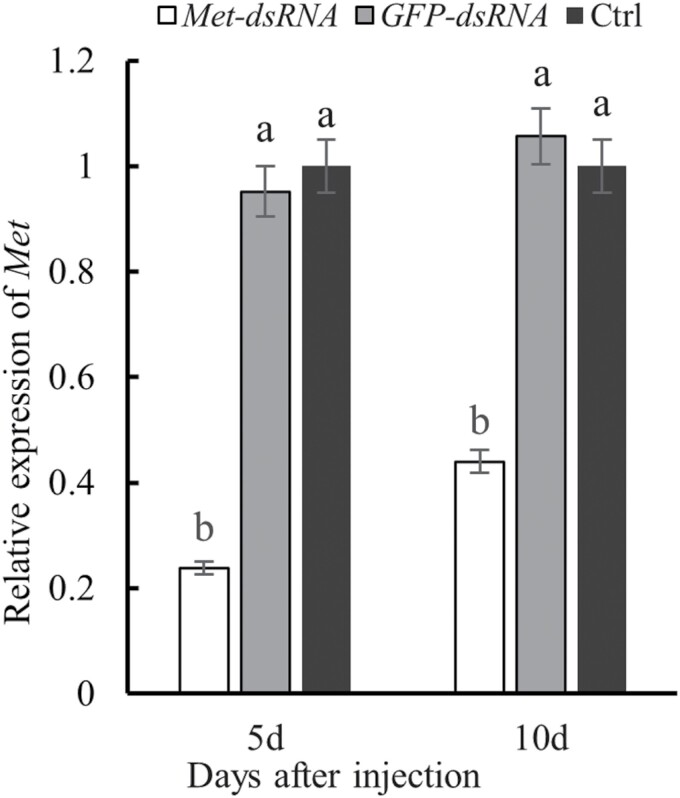
The relative expression levels of *CsMet* in *Coccinella septempunctata* at 5 and 10 d after dsRNA injection. Note: Ctrl, noninjected control. Data are mean ± SD. Columns labeled with different letters indicate significance at *P *< 0.05 using the LSD test.

*CsMet* expression decreased significantly after females were injected with *CsMet*-dsRNA (Fig. 7). At 5 d after injection, *CsMet* expression was decreased by 75.05% in females treated with *CsMet*-dsRNA as compared to the control group injected with *GFP*-dsRNA (*F* = 225.95, *df* = 1,2, *P *= 0.004). At 10 d after injection with *CsMet*-dsRNA, *CsMet* expression decreased by 58.38% as compared to the group injected with *GFP*-dsRNA (*F* = 39.43, *df* = 1,2, *P* = 0.024). There was no significant difference in *CsMet* expression between the controls that were either injected with *GFP*-dsRNA or not injected (Ctrl) (Fig. 7). These results indicate that injection with *CsMet*-dsRNA silenced the expression of *CsMet*.

#### *Effect of* CsMet*-dsRNA on ovary development in* C. septempunctata

**Fig. 8.**
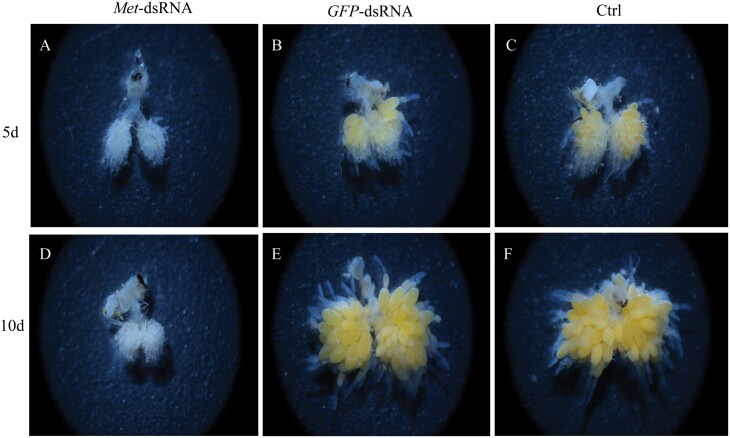
Ovary development in *Coccinella septempunctata* after dsRNA injection. Panels A–C) Ovaries of females at 5 d after microinjection with *CsMet*-dsRNA and *GFP*-dsRNA injection. Panels D–F) Ovaries of females at 10 d after *CsMet*-dsRNA and *GFP*-dsRNA injection, and the Ctrl represents ovary development in noninjected females.

**Fig. 9.**
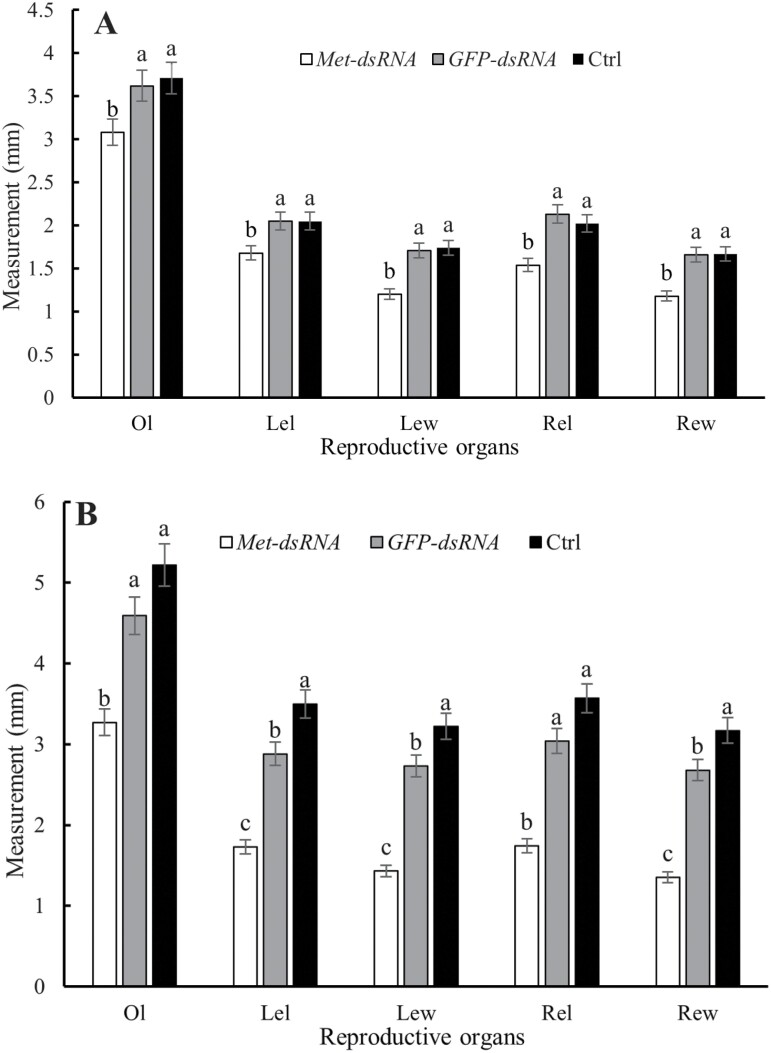
Ovary development in *Coccinella septempunctata* after *CsMet*-dsRNA injection. Note: Ctrl, noninjected control. Panels A) Ovary measurements 5 d after microinjection; B) ovary measurements 10 d after treatment. Abbreviations: Ol, ovary length; Lel, left egg-chamber length; Lew, left egg-chamber width; Rel, right egg-chamber length; and Rew, right egg-chamber width. Data are mean ± SD. Columns labeled with different letters indicate significance at *P *< 0.05 using the LSD test.

Dissection of *C. septempunctata* revealed that ovary development was significantly delayed in females injected with *CsMet*-dsRNA as compared to females injected with *GFP*-dsRNA (Fig. 8). Females injected with *GFP*-dsRNA had more mature eggs than those injected with *CsMet*-dsRNA, and most of the eggs in the latter group had less yolk deposition (Fig. 8). Measurements of ovary length, left egg-chamber length, left egg-chamber width, right egg-chamber length, and right egg-chamber width at 5 d after injection with *CsMet*-dsRNA were 14.92%, 18.05%, 29.82%, 24.14%, and 28.92% lower, respectively, than the group injected with *GFP*-dsRNA (Fig. 9A), and the differences were significant. Ovary length, left egg-chamber length, left egg-chamber width, right egg-chamber length, and right egg-chamber width at 10 d after injection with *CsMet*-dsRNA were 28.76%, 39.93%, 47.62%, 42.76%, and 49.63% lower, respectively, as compared to the *GFP*-dsRNA injection group (Fig. 9B), and the differences were significant. There were no significant differences in ovary development at 5 d postinjection with *GFP*-dsRNA and the noninjected control (Ctrl); However, at 10 d postinjection with *GFP*-dsRNA, ovary development was slower and fewer eggs were produced as compared to the noninjected control group.

#### *Effect of CsMet-dsRNA injection on egg production by* C. septempunctata

**Fig. 10.**
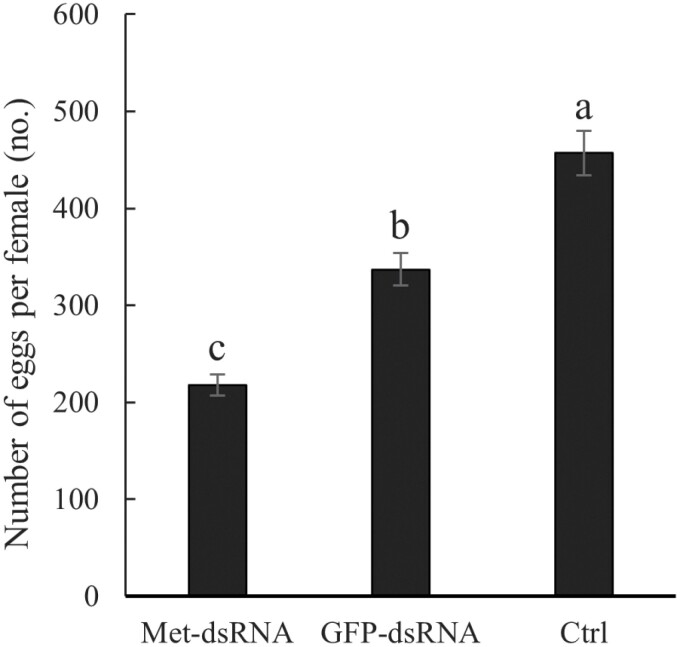
Fecundity of *Coccinella septempunctata* after *CsMet*-dsRNA injection. Note: Ctrl, noninjected control. Data are mean ± SD. Columns labeled with different letters indicate significance at *P *< 0.05 using the LSD test.

Egg production by individual females injected with *CsMet*-dsRNA was measured (Fig. 10). The average number of eggs produced per female injected with *CsMet*-dsRNA was 218, whereas mean egg production per female in the noninjected control and females injected with *GFP*-dsRNA was 457 and 337, respectively. Egg production by *C. septempunctata* injected with *CsMet*-dsRNA was significantly lower than females injected with *GFP*-dsRNA and the noninjected control (*F* = 41.07, *df* = 2,4, *P* = 0.002).

## Discussion

JH is an important insect gonadotropin that regulates reproduction (Zhou et al. 2012), and *Met* encodes a receptor in the JH signaling pathway. *Met* belongs to the 2 PAS transcription factor family (Ashok et al. 1998), which includes a basic DNA-binding region, a helix-loop-helix structure, 2 spatially variable PAS region (PAS-A and PAS-B), and a C-terminal PAC. The 2 protein domains of *CsMet* in *C. septempunctata* contained the bHLH domain and PAS repeat and were consistent with the sequences reported by Ashok et al. (1998). *Met* has been cloned from *Blattella germanica*, *Diploptera punctata*, *H. axyridis*, *Plutella xylostella*, *Nilaparavata lugens*, and *A. ipsilon* (Lozano and Belles 2014, Marchal et al. 2014, Yao 2015, Peng et al. 2020, Gassias et al. 2021, Han et al. 2022). Most insects have the same *Met* structural domain, but some species have different PAS motifs, indicating that Met may have different functions in different species. The phylogenetic analysis showed that *CsMet* was closely related to *HaMet* in *H. axyridis*. RNA interference technology found that *Met* knockdown inhibited the transcription levels of vitellogenin (Vg) and vitellogenin receptor (VgR) genes, oocyte maturation and oogenesis in Hemiptera, Lepidoptera, Coleoptera (Gijbels et al. 2019, Zhang et al. 2019, Miao et al. 2020, Huangfu et al. 2021).

Real-time quantitative PCR revealed that *CsMet* is expressed in all developmental stages of *C. septempunctata*. In larvae, the highest expression levels were observed in third-instar larvae, which may reflect the rapid growth in this developmental stage. The rapid decrease in *CsMet* expression levels in fourth-instar larvae suggests that *CsMet* may be involved in metamorphosis and development of ladybug larvae into pupae. This speculation is consistent with results obtained for *Chilo suppressalis* (Miao et al. 2020), *Helicoverpa armigera* (Ma et al. 2018), and *Sitodiplosis mosellana* (Cheng et al. 2020b) and indicates that JH regulates metamorphosis through the *Met*-encoded receptor. When *C. septempunctata* entered the pupal stage (2-d-old), *CsMet* expression was significantly upregulated, and it has been speculated that elevated expression in pupae may be associated with metamorphosis and wing dimorphism (Kayukawa et al. 2014, Lozano and Belles 2014).

The expression of *CsMet* in *C. septempunctata* was relatively low from eclosion to preoviposition (1–10 d following eclosion). When female ladybugs entered the peak ovulation period (20–30 d after eclosion), *CsMet* expression significantly increased. The high expression levels of *Met* in adults may help regulate reproductive development and life span (Smykal et al. 2014, Hejnikova et al. 2016), whereas the physiological processes governed by *Met* remain unclear. Furthermore, *CsMet* expression was highest in the fat body of *C. septempunctata* adults, which is consistent with expression in *D. punctata* (Marchal et al. 2014) and *Locusta migratoria* (Guo et al. 2014). Interestingly, the *CpMet* expression levels in *Culex pipiens palens* were higher in the ovaries as compared to fat body (Zhou et al. 2021).

Other researchers have used RNAi to silence *Met* genes and evaluate their roles in yolk deposition, ovary development, and reproductive ability. Knockdown of *Met* expression in *T. castaneum* interfered with the synthesis of yolk proteins and egg production (Parthasarathy et al. 2008, 2009). Similarly, silencing *Met* in *D. punctata* interfered with oocyte growth and the production of vitellogenin, thus impacting ovary development (Marchal et al. 2014). Knockdown of *Met* expression also inhibited ovary development In *Pyrrhocoris apterus* (Smykal et al. 2014). In this study, RNAi-mediated suppression of *CsMet* expression levels in female adults of *C. septempunctata* resulted in delayed ovary development. Female insects were able to mate and deposit eggs, but the yield of eggs was significantly lower than the controls. In our study, we used microinjection to introduce dsRNA into ladybirds, and this caused some damage to adult females due to the difficulty of the microinjection process. Consequently, egg production in females injected with *GFP*-dsRNA was lower than noninjected controls, and this difference was likely due to mechanical damage, and *GFP*-dsRNA can elicit an immune, antiviral response and thus elicit an elevated level of stress, or GFP siRNA causes an off-target effect. Moreover, the preoviposition and oviposition periods of *C. septempunctata* are relatively long, and the efficacy of *CsMet* suppression by RNAi likely decreases over time.

In summary, this study describes the cloning and expression of *CsMet.* The regulatory effect of *CsMet* on *C. septempunctata* reproduction was confirmed using RNAi technology, which is important in the context of understanding the molecular basis of JH signaling pathways in the regulation of insect reproduction. Furthermore, this study provides a foundation for future studies aimed at improving artificial diets for ladybirds.
